# Let’s talk about AIDS, baby! Critiquing the HIV and AIDS Act, 2017 in India through a reproductive justice framework

**DOI:** 10.1093/medlaw/fwaf034

**Published:** 2025-09-22

**Authors:** Apoorva Nangia, Jwalika Balaji

**Affiliations:** Supreme Court of India, New Delhi, India; National Law School of India University, Bengaluru, Karnataka, India; National Law School of India University, Bengaluru, Karnataka, India; Vidhi Centre for Legal Policy, New Delhi, India; University of Oxford, Oxford, United Kingdom

**Keywords:** anti-discrimination law, gender and sexuality, India, intersectionality, reproductive justice, HIV AIDS Act 2017

## Abstract

The HIV and AIDS Act was enacted in India in 2017 to protect the right to privacy, bodily autonomy, and non-discrimination of Persons Living with HIV (PLHIV). Prior to the Act, HIV/AIDS-related jurisprudence in India developed largely through judicial decisions on privacy, health care, queer rights, and marriage. This jurisprudence stigmatised PLHIV and constructed them as threats to society. The Act sought to respond to such characterisations and affirmed several rights of PLHIV that were previously denied. This article uses the reproductive justice framework to analyse the scope and potential of the Act in furthering sexual and reproductive freedoms of PLHIV and their partners. The article argues that despite the various rights and anti-discrimination protections under the Act, the law is insufficient to address the systemic and structural issues affecting PLHIV. The Act is premised on the problematic assumption that PLHIV are ‘risky’ sexual subjects, which affects the construction of rights and benefits. Furthermore, the Act does not, and arguably cannot, holistically address the problems faced by PLHIV in sexual, marital, and familial relationships. These constraints are a crucial reminder of the larger limitation of law as a blunt policy tool that is unable to comprehensively address complex socio-legal issues.

## I. INTRODUCTION 

The Human Immunodeficiency Virus and Acquired Immune Deficiency Syndrome (Prevention and Control) Act (‘the Act’) was enacted in India in 2017 with the aim of preventing and controlling the spread of HIV and AIDS.^1^ The Preamble of the Act states that it is to protect the human rights of persons who are HIV-positive (‘Persons Living with HIV’ or ‘PLHIV’) or vulnerable to the virus or the syndrome. It also recognises the need to protect the rights of healthcare providers and others related to PLHIV. The Act follows the approach laid down in the UN General Assembly’s Declaration of Commitment on Human Immunodeficiency Virus and Acquired Immune Deficiency Syndrome (2001) to combat the issue in a comprehensive manner.^2^ The Act responded to the lacuna and problems in India’s legal treatment of PLHIV by, for the first time, protecting against discrimination, empowering PLHIV to manage their own health care, and providing them with allied rights to live a life with dignity.

In this article, we argue that while the Act addresses various issues in the erstwhile jurisprudence and legal discourse on HIV by granting individual-centric liberal rights to PLHIV, the Act does not account for PLHIV as sexual and relational beings. The way in which the law seeks to address stigma and discrimination does not adequately engage with their systemic causes, especially in the context of familial, marital, and sexual relationships, which results in the denial of sexual and reproductive freedoms. The purpose of this argument is to show that even progressive legislation that secures individual rights is, in itself, insufficient to secure justice in the absence of social and cultural change. We recognise that the law can only go so far; the point of the article is to argue that progressive legislation cannot and should not be the only answer to broader social problems; paradoxically, it may even narrow down the space available for non-legal engagement. Therefore, the aim is to highlight the limitations of the law.

The article aims to fill a gap in scholarship surrounding the Act. While scholars have approvingly documented the various rights and protections granted by the Act, there is no comprehensive analysis and critique of the Act from a reproductive justice perspective, which would enable viewing PLHIV as relational and sexual beings. While the relational model encourages us to see individuals as situated within networks of care and responsibility, the reproductive justice framework goes further by demanding structural transformation—across health, education, and legal institutions—to create conditions for exercising bodily autonomy. This is what makes the reproductive justice framework a particularly compelling lens for analysing the HIV/AIDS Act.

The reproductive justice framework is built on the reproductive rights framework, but goes beyond that to achieve an intersectional view of justice. It focuses on marginalised communities’ access to reproductive health, enabling them to make decisions in a manner that both respects their autonomy and their personal, familial, and social relationships.^3^ Reproductive justice is concerned with people’s sexual, physical, mental, and emotional well-being, and seeks to empower people to take charge of decisions relating to their own bodies, sexualities, and reproductive and allied interests. Reproductive justice is methodologically intersectional, acknowledges and affirms the relationality of human beings, and calls for paying attention to individuals vis-à-vis the broader social forces acting upon and alongside them.^4^ Finally, reproductive justice is also concerned with structural change, borrowing from its activist roots, to argue that the way to achieve reproductive justice must necessarily extend beyond the law and to social, cultural, political, and economic change.^5^

To give an example, abortion as viewed through the reproductive justice framework would not be located within the liberal rights framework, that is, the pro-choice versus pro-life approach.^6^ Rather, from the reproductive justice framework, we would not only look at health concerns, but ask the pregnant person how they view themselves, the pregnancy, and their relationships with those around them. We would trace the ways in which reproductive autonomy (especially of those from intersectionally marginalised groups) has been regulated both socially and through the law. Ultimately, we would seek to empower the pregnant person to take the best and most informed decision after considering all these factors, neither judging them for their decision nor controlling the outcome by other means.^7^ Such a framework would call for strengthening health and social welfare infrastructure, enabling both medical and social support for the pregnant person to effectively take a decision and not face adverse consequences for the same.

In the specific case of HIV, the framework gives us a metric to inquire into the law’s awareness of social realities and its success in empowering PLHIV in light of the same. Further, it allows us to move beyond the dualistic understanding of individual rights versus duties/obligations and instead explore the legal, ethical, and practical nuances and concerns of various stakeholders. It contests the black and white approach of the law by allowing us to understand and acknowledge conflicting concerns as simultaneously valid and demanding legal and/or non-legal engagement. We will use the reproductive justice framework in this article to analyse the provisions of the Act and the broader jurisprudence on HIV/AIDS. The scope of legal analysis is restricted to Indian legislation and jurisprudence.

In this article, we will: First, trace the novel legal protections offered by the Act to PLHIV. This includes protection against discrimination, recognition of PLHIV’s autonomy, and placing responsibility on certain stakeholders and government authorities to realise the rights of PLHIV and create the necessary infrastructure.

Second, locate the Act in Indian jurisprudence on HIV/AIDS in the areas of medical confidentiality and privacy, litigation for queer rights, and marriage and divorce. We argue that the Act responds positively to issues of privacy by recognising PLHIV as equal citizens, and protects activists and educators from criminal sanctions. However, the Act does not sufficiently engage with questions of criminal laws that impact PLHIV and laws that govern their sexual and marital relationships.

Third, critique the Act from a perspective informed by reproductive justice. We are using this framework to analyse the foundations and principles of the Act and to understand the social life of the law. We argue that the Act is limited in its approach to PLHIV’s sexual health and inter-personal relationships, and that it does not address broader social issues, both at a legal and practical level. We argue that the Act unilaterally burdens PLHIV with the duty of preventing transmission, while ignoring the complex socio-cultural factors that govern safe sex practices, condom usage, disclosure, and more broadly, the sexual well-being of all individuals. The Act has a limited and conventional understanding of relationships in general, and sexual relationships in particular, and fails to sufficiently account for the interests of the partners of PLHIV in some situations.

The aim of the article is not to suggest that the deficiencies of the Act can be rectified through legal redressal of these issues. Rather, the aim is to show how the Act provides definitive answers to certain concerns that can foreclose debate and block the imagination of more comprehensive solutions outside the law. The Act is simply limited because it is part of the law, which is inherently a blunt and limited tool to address complex socio-legal issues. This means that some issues can only be engaged with non-legally, and the article aims to trace the limits of the law in responding to and empowering PLHIV to take decisions for their and their partners’ well-being.

## II. TRACING THE FEATURES OF THE ACT

In this section, we outline the important and novel features of the Act. It is a progressive legislation in some respects, and provides for the following safeguards and protections to PLHIV: first, it prohibits discrimination against PLHIV and associational discrimination; second, it recognises the medical autonomy of PLHIV; third, it places PLHIV in control of their sensitive information; fourth, it creates obligations on several actors; and fifth, it sets up the institution of an Ombudsman to provide remedy against discrimination.

First, the Act prohibits discrimination against protected persons, that is, PLHIV and those who reside or cohabit with them or did so previously.^8^ Discrimination is defined as the imposition of burdens, disability, or disadvantage, or the withholding of benefits from PLHIV on HIV-related grounds.^9^ Discrimination is prohibited in employment, housing, health care, educational establishments, shops and services, public infrastructure, and insurance. Isolation or segregation of protected persons is also prohibited by the Act.^10^ Spreading hatred towards PLHIV is considered an offence under the Act, with a corresponding penalty.^11^

Secondly, the Act recognises the medical and bodily autonomy of PLHIV to take their own healthcare decisions. It mandates informed consent before undertaking HIV tests or other medical interventions.^12^ Exceptions are minimal and for specific purposes like epidemiology, screening at a blood bank, determination of court issues, and medical research.^13^ Further, no HIV-positive pregnant woman can be subject to sterilisation or abortion without their consent.^14^ The Act also affirms that every person in the care or custody of the State shall have the right to HIV-prevention measures, counselling, care, and treatment. Such persons include convicts (including under laws relating to sex work), undertrials,^15^ and children in conflict with the law.^16^

Third, the Act classifies HIV-related information as sensitive, and enables PLHIV to decide whether, when, and to whom they wish to disclose it. It simultaneously places a duty on PLHIV, post-counselling, to take reasonable precautions to prevent transmission to sexual partners or to persons with whom they share needles.^17^ However, no person can generally be compelled to disclose their HIV status, with a few limited exceptions. The Act also places a broad prohibition on health care providers from disclosing HIV status to partners. Only physicians and counsellors are allowed to do so, and only where they suspect that there is a serious risk to the partner due to non-disclosure by the PLHIV. Even in that circumstance, the physician or the counsellor is required to inform the PLHIV of their intention to inform the partner. There is a special exception carved out for women, for both self-disclosure and by third parties, such that disclosure is not mandated if there is a reasonable apprehension that it could result in violence, abandonment, or have a severe effect on the mental and physical health of the woman, her children, family, and those close to her.^18^ In the same vein of navigating the social repercussions of disclosure, the Act ensures that in any proceeding related to a PLHIV, anonymity shall be maintained as far as possible in the judgment as well as in general reporting.^19^

Fourth, the Act imposes specific obligations on certain actors and authorities. It requires establishments to ensure that there is adequate data protection of PLHIV’s data. It also requires them to undertake precautions if the nature of the occupation exposes workers to contracting HIV-AIDS.^20^ The Act places an obligation on the central and state governments to, as far as possible, set up diagnostic and infection-management facilities. It requires the government to facilitate access to welfare schemes and formulate sensitive and non-stigmatising awareness programmes for PLHIV.^21^

Lastly, the Act sets up an Ombudsman to decide complaints of discrimination on HIV-related grounds. It lays down the procedure for the functioning of the Ombudsman as well.^22^

In the next section, we contextualise these provisions of the Act, mapping how they respond to certain social, legal, and political concerns that arose from existing perceptions, both legal and non-legal, of PLHIV. We argue that the Act responds positively to issues of privacy by recognising PLHIV as equal citizens, and protects activists and educators from criminal sanctions. However, the Act does not sufficiently engage with questions of criminal laws that impact PLHIV and laws that govern their sexual and marital relationships.

## III. LOCATING THE ACT IN INDIAN JURISPRUDENCE ON HIV/AIDS

In this section, we examine the jurisprudence and attitude of the law towards HIV/AIDS and PLHIV before the Act came into force. Through this exercise, we seek to identify the similarities and divergences of the Act from erstwhile legal approaches. This locates the Act in prior jurisprudence that informs its provisions and shows a certain change in the legal discourse on HIV/AIDS. We broadly examine the Act through the lens of four issues: privacy and health care, queer litigation and activism, criminal law, and marriage and divorce.

### A. Privacy and health care

There are three ways in which the Act safeguards the privacy of PLHIV, vis-à-vis health care and medical treatment. Firstly, it protects the informational privacy^23^ of PLHIV by broadly giving them control over the disclosure of their HIV status, with limited exceptions. Secondly, it mandates informed consent before HIV testing, and also builds in pre-test and post-test counselling as part of the informed consent conditions. Thirdly, the Act refutes strategies of ostracisation based on harmful stereotypes and instead reaffirms the rights of PLHIV to access public goods, services, places, and infrastructure without discrimination.

#### 1. Informational privacy

PLHIV have been viewed as risks *in rem*, and the fear of transmission has been used to permit disclosure without informed consent. One of the first HIV-related decisions in India was in *Mr X v Hospital Z*,^24^ wherein the hospital disclosed the HIV-positive status of Mr X to his fiancée, Ms Y. The marriage was called off and Mr X was ostracised by his community. He challenged the disclosure as violating the duty of confidentiality and his privacy. The Supreme Court of India held that the disclosure was legally valid and that privacy could be restricted for the protection of public health and to protect the ‘life of an innocent woman’.^25^ The Court also stated that the right to marry of PLHIV was suspended as marriage is for a ‘healthy body’.^26^ While the statements on marriage were later held to be obiter and not legally binding,^27^ the case set a precedent for enabling doctors to share medical information of PLHIV with potential spouses. However, these requirements do not account for the privacy and decisional autonomy of PLHIV (and their partners).

As a response to such denial of decisional autonomy, Sections 8 and 9 of the Act redress these issues by preventing third parties, including doctors, from disclosing the HIV status of a person without their informed consent. PLHIV cannot be compelled into disclosure without a court order. Both these provisions secure the informational privacy of PLHIV to control what information about them is disseminated, to what extent, and to whom. Informational privacy has been recognised and protected by the Supreme Court of India in *KS Puttaswamy*, which was decided in the same month as the passing of the Act, as a facet of the right to life under Article 21 of the Constitution.^28^ The Act attempts to strike a balance between informational privacy and the interests of potential partners by enabling doctors to disclose only in extreme situations when the PLHIV has been counselled and still does not disclose their HIV status to their partner(s).

#### 2. Informed consent

Concerns of transmission within married couples were reflected in the ‘Compulsory Pre-Marital HIV Test and Other Measures Bill 2010’ that sought to make pre-marital testing mandatory.^29^ Reproductive justice scholar Dipika Jain argues that while such a policy crucially recognises that transmission overwhelmingly occurs within heterosexual marital sex, she critiques the policy for violating privacy and informed consent.^30^ Jain also argues that such policies do not account for counselling, which is essential to enable PLHIV and their partners to understand, cope with, and manage the condition.^31^ The Act, on the other hand, recognises that mandatory testing is likely to lead to State coercion and surveillance over persons. Further, mandatory testing takes away decision-making from persons about their own health, bodies, and information. The Act therefore responds to these critiques by mandating informed consent for HIV-testing and pre-test and post-test counselling of the person being tested or their representative.^32^

#### 3. Anti-discrimination and prohibiting ostracisation

Medical professionals and courts previously encouraged the physical isolation and segregation of PLHIV as a way to promote public health.^33^ The Goa Public Health (Amendment) Act, 1987 was a law under which AIDS activist Dominic D’Souza was forcefully isolated in his home. The Bombay High Court upheld the validity of such action by reasoning that while isolation has serious consequences on liberty and social and economic matters, these must give way to ‘public interest’. It held that isolation is in the interest of society and of the PLHIV, as a way to ‘*save him against himself*’.^34^ The Court characterised PLHIV as ‘desperate and los[ing] all hopes of survival’.^35^ A similar trend of courts ‘saving’ PLHIVs is visible in the Bombay High Court’s unpublished order to mass arrest female sex workers due to fear of transmission.^36^ It justified mandatory testing, raids, and detention as a way to curb the spread of HIV.^37^ The Act combats these stereotypes and unjustified fears by expressly reaffirming the human rights of PLHIV in the Preamble and cementing their right to access public places, education, employment, and housing without discrimination or ostracisation.^38^

Through judicial decisions, it is visible how, in the law, PLHIV were viewed as dangers and risks to society. The Act counters this approach to public health and recognises the rights of PLHIV in a way that affirms their medical autonomy and informational privacy, combats stigma and stereotypes, and enables them to access substantive goods and services without discrimination.

### B. Litigation for queer rights

The HIV/AIDS battle has historically been entangled with litigation and activism for queer rights in India. Section 377 of the erstwhile Indian Penal Code, 1860, which criminalised any form of ‘voluntary carnal intercourse against the order of nature’ was effectively a prohibition on same-sex sexual intercourse.^39^ Even though the provision did not lead to a large number of prosecutions, it was an effective tool for the police to harass, threaten, blackmail, and even assault queer persons in some circumstances.^40^ This provision had a chilling effect on non-governmental organisations (NGOs), activists, and others working to address the spread of HIV/AIDS, mainly among transgender persons and men who have sex with men (MSM).^41^ These NGOs and activists were actively targeted by the government, which denied that HIV/AIDS was an issue in India and refused to acknowledge its transmission among gender and sexual minorities.^42^

Post-liberalisation in the 1990s, international donors who conceived of India as a ‘sexually repressed’ society made grants to the government on the condition that it adopt a humane and non-discriminatory approach to combat HIV/AIDS.^43^ This led to the birth of several, mostly foreign-funded NGOs that sought to address HIV prevention within the queer community. Two such NGOs—the AIDS Bhedbhav Virodhi Andolan (a non-funded NGO) in 1994 and Naz Foundation (a foreign-funded NGO) in 2001—filed petitions before the Delhi High Court challenging the constitutionality of Section 377.^44^ They argued that Section 377 affected the right to health care of queer persons by creating a chilling effect on disclosing HIV status and seeking health care.^45^ These petitions gained support from the National AIDS Control Organisation (NACO) as well.^46^

The Delhi High Court, in *Naz Foundation v Union of India*, read down section 377 to decriminalise private, same-sex, consensual sexual intercourse between adults.^47^ The Court recognised that MSM and transgender persons were subject to abuse under this provision, and that it led to discrimination in health care for HIV treatment.^48^ The Court reiterated India’s international commitments to equality in public health care, for which the reading down Section 377 was imperative.^49^ Unfortunately, this judgment was problematically set aside in appeal in 2013 by a division bench of the Supreme Court in *Suresh Kumar Kaushal v Naz Foundation* for a variety of reasons (which have been extensively critiqued).^50^ This again prompted concerns of backlash and harassment against queer individuals, as well as those involved in HIV/AIDS prevention efforts within the queer community.

Section 22 of the Act responds to this history and jurisprudence.^51^ It carves out an exception for activities undertaken by persons, establishments, or organisations working for the control and prevention of HIV to be exempted from criminal or civil liability. The illustrations clearly protect those engaged in providing information and condoms to MSM persons for the purpose of HIV control.^52^ The Act is thus sensitive to the history of HIV prevention and control efforts within the queer community, and recognises the importance of non-discrimination in health care.

Later, the Supreme Court’s decision in *Navtej Singh Johar v Union of India* in 2018^53^ (which overruled *Suresh Kumar Koushal*) reaffirmed the reasoning in *Naz*, and held that one of the main reasons, among others, for reading down Section 377 was to enable equality in sexual health care and treatment of HIV among queer and transgender persons.^54^ Therefore, the Act is now backed by a rich legal jurisprudence that supports access to care and treatment for all individuals irrespective of gender identity or sexual orientation. It allows NGOs and activists to function safely and effectively.

However, the most important critique of both *Naz* and *Navtej* is that they only decriminalise *private* consensual, same-sex intercourse,^55^ which offers no protection to sex (paid or unpaid) in the public sphere. Many queer persons do not have access to private spaces to engage in sexual activity. Further, queer sex workers use public spaces to solicit clients and engage in commercial sex work.^56^ The interplay of criminalisation of public sex and the spread of HIV in these spaces is not effectively addressed by the Act, as shown in the next section.

### C. Other criminal laws

There are three ways in which criminal laws intersect with the Act; however, the Act is found lacking in its engagement with them. First, the Act fails to acknowledge and grapple with the effects of criminalisation of sex work, which drives sex work underground and thereby creates a chilling effect on accessing HIV-related health care. Second, while the Act recognises the gendered impact of disclosure and exempts disclosure of HIV-positive status for women, it does not address how this would affect provisions that criminalise negligent and malicious transmission of HIV. Third, the Act is not useful when considering the interplay between disclosure, consent, and sexual harassment/assault laws.

#### 1. Criminalisation of sex work

The Immoral Traffic Prevention Act, 1956 criminalises various aspects of sex work such as maintaining brothels, soliciting, and trafficking.^57^ While sex work is in itself not criminalised, it is stigmatised both under this law and by courts.^58^ In one case that sought complete decriminalisation of sex work, wherein one of the arguments pertained to HIV-prevention, the Gujarat High Court held that criminalisation in fact prevents the spread of HIV.^59^ It reasoned that legalising sex work as a means of livelihood would legitimise trafficking and exploitation, and characterised the profession itself as causing HIV, other sexually transmitted diseases, and drug addiction.^60^

However, as was highlighted in *Naz*, criminalisation is counterproductive as it creates a chilling effect and instead drives marginalised groups underground, thereby depriving them of essential care and treatment for HIV. While Section 22 of the Act recognises this, it fails to address the problem of criminalisation and provides protection to actors only to the limited extent of activities that reduce or prevent transmission, such as HIV-related and safe sex education, provision of condoms and sexual safety equipment, and provision of injection safety requirements.

#### 2. Negligent and malignant transmission of diseases

Further, criminal laws create penal sanctions for the transmission of HIV through sex. Sections 269 and 270 of the erstwhile Indian Penal Code, and Sections 271 and 272 of the Bharatiya Nyaya Sanhita, 2023 penalise negligent and malicious transmission of dangerous diseases. However, such criminal laws are counterproductive when the source of transmission is unclear, as is often the case in marital relationships. Women are often blamed for being the source of the disease, despite being unaware of its existence or being infected by their husbands.^61^ However, women rarely employ these provisions against their husbands as they are not informed of their HIV status, nor do they have the resources to pursue litigation.^62^ Hence, criminalisation of transmission has a gendered impact that disadvantages women.

The Act acknowledges the gendered impact of disclosure to an extent. Sections 9 and 10 provide for disclosure by healthcare professionals and uphold the duty of PLHIV to prevent transmission.^63^ However, the provisos to both sections exempt women with HIV/AIDS and their healthcare professionals from disclosing their HIV status to their partners if there is reasonable apprehension that such disclosure may result in violence, abandonment, or actions that severely impact the mental or physical health of the woman, her children, her relatives, or someone close to her. However, the interplay of such an exemption with laws that criminalise negligent and malicious transmission is unclear as it is uncertain whether the exemption constitutes a defence in criminal law.

#### 3. Interplay with consent and sexual assault provisions

HIV disclosure does not feature in the sexual assault provisions as a factor that determines the negotiation of consent. In a rape case before the Delhi High Court, the Court delved into transmission during sexual assault and relied on jurisprudence from other countries to hold that transmission of HIV is an aggravating factor in sentencing.^64^ It also held that sexual activity by PLHIV is not criminalised if the partner has consented.^65^ However, it is unclear as to what the required standard and quality of such consent is. Knowledge of HIV status, treatment, and awareness of safe-sex practices all play a role in determining consent to specific sexual activities.^66^ However, the Court in *Sabhajeet* failed to clarify what the consequences would be upon the legal understanding of consent if such HIV status was not disclosed. The Court failed to nuance its understanding of consent, which leaves open questions on the applicability of sexual assault laws when a PLHIV knowingly does not disclose their status to a sexual partner.

More generally, the HIV/AIDS jurisprudence in criminal law has not been addressed in the Act in detail or depth. It remains to be seen whether its progressive language and discourse will be used by courts while dealing with cases of transmission by PLHIV or whether the jurisprudence of stigma and risk will continue.

### D. Marriage and divorce

Various legislations on marriage in India provide for divorce by a spouse when the other spouse is suffering from a venereal disease.^67^ Courts have recognised that HIV/AIDS is a venereal disease as it is transmissible through sexual intercourse and hence, divorce can be granted on this basis.^68^ To enable the court to determine the presence of HIV in either spouse, it can direct mandatory testing.^69^ The Act also follows this framework by doing away with the informed consent requirement when the court directs it.^70^

Another ground that has been deployed for divorcing spouses with HIV is cruelty. In a divorce petition by a husband, the Punjab and Haryana High Court held that since his wife was suffering from HIV, which is an incurable and communicable disease, the husband was entitled to dissolution of marriage on the grounds of cruelty.^71^ While the husband had established other grounds for cruelty, the characterisation of HIV as a form of marital cruelty stigmatises PLHIV and the disease itself. The term ‘cruelty’ also carries with it an attribution of fault,^72^ which may not always be the case with the way HIV is transmitted. Although the Act is progressive in many aspects, the stigma attached to PLHIV and viewing a marital relationship with PLHIV as a case of ‘cruelty’ per se is a characterisation that has not been (and perhaps cannot be) addressed by the Act alone.

### E. Concluding summary

To sum up, many provisions of the Act can be correlated with redressing the deprivation of the rights of PLHIV within an erstwhile legal discourse of risk, stigma, and exclusion. The Act primarily secures the rights of privacy, bodily and decisional autonomy, medical confidentiality, non-discrimination, and access to health care. However, some parts of existing jurisprudence have not been dealt with very clearly, or at all, under the Act. The Act does not necessarily mark a shift or break in the legal discourse of stigma in all respects, as such stigma cannot be located in a singular source but is more fragmented and pervasive across the legal body on sex, marriage, disease, and health care.

## IV. CRITIQUE OF THE ACT FROM A REPRODUCTIVE JUSTICE FRAMEWORK

The Act is generally progressive in being one of the few legislations that acknowledges and redresses discrimination by private individuals in employment, housing, education, medical care, and insurance.^73^ PLHIV have used provisions of the Act to seek relief from wrongful termination due to HIV status. In one such case, the petitioner was wrongfully terminated for taking ‘unauthorised leave’.^74^ He took such leave due to medical reasons associated with HIV. Further, he argued that the employer was aware of his HIV status and dismissed him on that basis. While the Allahabad High Court did not entertain the writ petition as it was against a private employer, it directed the petitioner to approach the Ombudsman under the Act.^75^ In another case where the government provided compassionate appointment to a woman and subsequently terminated her on discovering her HIV status, the Madras High Court held that the employer should provide alternative employment to PLHIV that is commensurate with their medical condition, instead of dismissing them from service.^76^

While the law has accounted for the discrimination faced by PLHIV in the public sphere, the Act fails to address issues faced by PLHIV in their personal, sexual, and familial lives. *X v Z* is one of the most prominent cases in AIDS jurisprudence in India.^77^ The Supreme Court commented on the nature of those who suffered from AIDS:

“AIDS” is the product of undisciplined sexual impulse. This impulse, being a notorious human failing if not disciplined, can afflict and overtake anyone howsoever high or, for that matter, how low he may be in the social strata. The patients suffering from the dreadful disease “AIDS” deserve full sympathy. They are entitled to all respect as human beings. Their society cannot, and should not be avoided, which otherwise, would have a bad psychological impact upon them. They have to have their avocation. Government jobs or service cannot be denied to them as has been laid down in some American decisions. But “sex” with them or the possibility thereof has to be avoided as otherwise they would infect and communicate the dreadful disease to others. The Court cannot assist that person to achieve that object.^78^

The underlying narrative about PLHIV is that they must abstain from sex, and that they are not acceptable sexual partners—their ‘disease’ must define and control their sexual desire. PLHIV are not viewed as individuals who can engage in healthy and safe sexual relationships. The Act does not explicitly engage with this view and takes some definitive positions, which may be harmful to PLHIV and their partners. The reproductive justice framework is useful to critique the Act on three pillars:

First, the framework enables us to recognise ‘reproductive oppression’^79^ and ‘reproductive suffering’. Reproductive oppression refers to the double-bind faced by marginalised groups through control and exploitation of their bodies, reproduction, and sexuality by families, communities, institutions, and society, which renders them even more vulnerable and prone to oppression.^80^ Reproductive suffering refers to barriers or suffering experienced by persons facing similar issues and concerns, but who may have the means and the wherewithal in some form to minimise the consequences of these issues.^81^ Second, the reproductive justice framework enables us to capture these lived realities and locate them within law and policy. Third, the reproductive justice framework enables analysis both from an inter-personal and community lens (relational) as well as from a systematic lens. It equips us with the tools not only to address concrete, minute problems but also to challenge and mediate our interactions with structural barriers of oppression.

From this perspective, we discuss four larger, conceptual issues in the Act emanating from structural barriers: (i) unilateral burden on PLHIV to prevent transmission; (ii) lack of engagement with larger structural issues governing disclosure and safe-sex practices; (iii) lack of protection for all possible partners of PLHIV; (iv) perpetuation of stigmatic categorisations of PLHIV.

### A. Unilateral burden on PLHIV to prevent transmission

First, the Act does not engage with the politics of safe sex practices. The entire burden is on the PLHIV to take reasonable precautions to prevent transmission of HIV, especially through sexual contact.^82^ Section 10 of the Act states,

Every person, who is HIV-positive and has been counselled in accordance with the guidelines issued or is aware of the nature of HIV and its transmission, shall take all reasonable precautions to prevent the transmission of HIV to other persons which may include adopting strategies for the reduction of risk or informing in advance his HIV status before any sexual contact with any person or with whom needles are shared with.^83^

While it is definitely important to create a legal duty that encourages curbing the transmission of HIV, the legal nature of the duty does not entirely account for the politics of safe sex practices. There is a limited exception that allows women to not disclose their HIV status during sexual contact if there is reasonable apprehension of violence, abandonment, or a negative effect on their health or their children and other loved ones.^84^ While this is an interesting way to legally immunise women from disclosure requirements, the overall legal duty on PLHIV may be quite difficult to actualise, given the complexity of safe sexual practices among different demographics, as evidenced below.

Studies have shown that husbands in heterosexual marriages in India dominate decision-making about condom usage due to a number of factors, such as men being the general decision-makers in households, the prevalence of intra-personal violence, and a lack of communication between partners about sex.^85^ Further, it is a common cultural belief in India (albeit a wrong one) that a married woman cannot be infected by her husband,^86^ and therefore women insisting on condom usage is viewed with suspicion.^87^

When it comes to non-usage of condoms by female sex-workers, some cite lack of knowledge on HIV prevention, while others stress the lack of access to free condoms.^88^ Some studies have found that brothel-based sex workers are able to practice and insist upon condom usage because organised groups of sex workers support each other when it comes to unwilling or violent clients.^89^ Non-brothel-based independent sex workers, on the other hand, are more vulnerable to exploitation. Some trans and MSM sex workers did not insist on condom use with regular male partners due to similar reasons as stated by married women: fear of violence, being thought of as promiscuous, and fear of stigma.^90^ Many transgender sex workers are often subject to forced sexual activity by police officers and local goons, and cannot control condom usage.^91^

Apart from identity-based factors such as gender, caste, class, and sexuality, other factors such as marital status, region, profession, and level of education and awareness also influence safe sex practices, and can lead to reproductive oppression for these groups. Their intersectional identities compound their vulnerabilities and hinder or prevent them from exercising agency in sexual interactions. These findings are important to understand the limits of imposing a legal duty to prevent transmission. They encourage thinking more carefully about how to engage with structural and cultural factors that govern the politics of condom usage and safe sex. The reproductive justice framework, therefore, would advocate for both legal and extra-legal acknowledgement of these aforementioned trends as the first step to realising the duty of reasonable precaution.

Further, the reproductive justice framework encourages us to see individuals as relational beings. While it is important to recognise the responsibility of PLHIV to take reasonable precautions to prevent the transmission of HIV, navigating sex is often a decision that partners take, or at least ought to take, together. While the Act cannot mandate the same (as is often the case when the law cannot step in to govern gender-equal decision-making within the household), this feature of social life is something that needs to be addressed through other means. Of course, these larger structural changes are not possible unless at least two basic factors change: first, there is comprehensive sex education that teaches sex as a shared, consensual, safe activity, and second, that there is a material and normative change in gendered power imbalances within and outside the household.

### B. The lack of engagement with larger structural issues governing disclosure and safe-sex practices

The Act, in general, fails to address the larger structural and systemic issues that lead to stigma and violence upon disclosure. The Act’s approach is largely to place responsibility on the PLHIV to prevent transmission through disclosure (subject to the exemption for women). Such a provision privatises and individualises the decision of disclosure, rather than understanding the decision in its social context. Many PLHIV do not disclose this information to partners due to the fear of divorce (as was seen in various cases above), removal from familial residence,^92^ or discrimination towards themselves and their family members.^93^ These concerns are especially exacerbated for women in their marital homes.^94^ However, publicly addressing and engaging with these issues is essential in the reproductive justice framework as it shifts the burden of freedom from individual rights to societal structures that hinder freedom.

The Act recognises the issue of imbalance in power between partners within sexual relationships. However, its solution is to exempt only women, wholly as a class, from the requirement of preventing transmission by sexual contact, if there is a reasonable apprehension that disclosure could result in violence or abandonment. This, however, has two issues—one, it frames only women as vulnerable within sexual relationships, while completely ignoring complex family dynamics, queerness, and other identity markers as factors influencing vulnerability, and two, the legal response to such violence completely forecloses the possibility of a discourse on attitudinal and social change regarding use of condoms and negotiating safe sex. The exemption from disclosure for women may provide a short-term legal solution to gendered violence, but it forecloses alternative ways of addressing the politics of safe sex in a more organic, social manner through activism and sex education. This may not be within the limits of the law; in fact, the reproductive justice approach recognises that not all solutions to socio-legal issues can be encapsulated within the law. This is the primary difference between the reproductive rights framework and the reproductive justice framework.

Reproductive Rights is a legal and advocacy-based model that … addresses the lack of legal protection and weak enforcement of laws to protect individual women’s reproductive choices regarding health care services.

Reproductive Justice is a movement-building and organizing framework that identifies how reproductive oppression is the result of the intersection of multiple oppressions and is inherently connected to the struggle for social justice and human rights. Reproductive justice argues that social institutions, the environment, economics, and culture affect each woman’s reproductive life.^95^

Applying this lens to HIV, the twin questions of disclosure and exemption from disclosure thus do not yield themselves to clear-cut legal ‘solutions’. When the Act takes a definitive position by exempting women from disclosure, it may be pre-emptively foreclosing dialogue on an important, multifaceted issue simply because now the issue seems to be definitively legally resolved. However, we must remember that the law only answers a very limited part of the problem, that is, legal culpability. The reproductive justice framework reiterates that comprehensive engagement with the question of power imbalance among partners requires a wider range of actors to step in, and consideration of varied social and political strategies. The social position of PLHIV and the systemic issues attached to disclosure mediated by gender, sexuality, ability, caste, religion, and marital status can be better addressed through dialogue, education, activism, and other efforts towards social and cultural change. Thus, the framework enables us to look beyond the letter of the law and consider the surrounding social circumstances as well—reflecting on the measures required alongside legislative reform. Further, a one-size-fits-all approach to the politics of disclosure would not be sufficient, and the nuances of intersectional identities should be taken into account at all levels—law and policy, social messaging, and activism and education.

### C. Lack of protection for all possible partners of PLHIV

The Act fails to sufficiently consider PLHIV as people who can have healthy and safe sexual relationships, and does not account for the safety of the partners of PLHIV. The Act protects the right of ‘partners’ to know [of the PLHIV’s HIV status] to some extent by enabling doctors to reveal the HIV-positive status of a person in specific circumstances.^96^ However, the definition of ‘partner’ is limited to spouses, *de facto spouses*, or those who are in a relationship in the nature of marriage with PLHIV.^97^ The Act privileges marital relationships to determine who is a partner. It does not extend the protections of the ‘right to know’^98^ to any sexual partner, which excludes non-marital sexual relationships among heterosexual couples, queer couples, and sex workers. These other sexual partners are only recognised in a very limited manner in section 10, where PLHIV are required to take ‘reasonable precautions’ to avoid the transmission of HIV. Differentiation in the quality of the safeguard, that is, a right to know, versus an expectation of ‘reasonable precaution’, which is based on the marital status of the partner, does not seem connected to the object of the Act, which is to prevent and control the transmission of HIV/AIDS. This places sexual partners other than spouses in a precarious condition, and we argue that privacy interests of PLHIV must be balanced with the ‘right to know’ of all partners’.^99^

The right to know is important to partners for several reasons. The Act does not recognise that knowledge of HIV-positive status is essential to enable all partners to negotiate sexual acts, protection, and care in a manner that accounts for their own health. For example, the pre-exposure prophylaxis (PReP) is a medicine that is often used by HIV-negative persons in sero-discordant relationships (where one of the partners is infected with HIV, but the other is not), beyond condom use, which can enable partners of PLHIV to engage in safe sexual intercourse with them. Such usage of PReP would not be possible without the person knowing of their partner’s HIV-positive status.^100^ While condom use as a means of precaution can be unilaterally employed by the PLHIV and hence satisfies the requirement under Section 10, it takes away decision-making from sexual partners as to how they wish to protect their own health. Hence, disclosure is essential for healthy, trusting, and consensual sexual relationships with HIV-positive persons. The distinction between reproductive oppression and reproductive suffering is yet again important here. The partners who are not heterosexual spouses deserve the right to know because they may undergo reproductive suffering with the threat of contracting HIV/AIDS. However, this suffering could be mitigated by the right to know, because that right would enable navigating sex with informed consent, using condoms, contraceptives, and other measures for safe sex, and even possibly getting on PrEP or similar medication.

The Act is also silent on counselling and empowering sexual partners, both generally and in sero-discordant relationships, to lead healthy sex lives and prevent the transmission of HIV. This gap indicates that the law does not typically envisage PLHIV to have HIV-negative partners or partners at all, let alone encouraging shared responsibility among the partners.

### D. Perpetuation of stigmatic categorisations of PLHIV

The issues above pertained primarily to accounting for the personal, familial, and sexual relationships and critiquing the Act for not being cognisant of complex inter-personal and social power dynamics. However, beyond the Act, a broader issue that exists in state policy and strategy is the categorisation of PLHIV as ‘risky’. The reproductive justice framework is useful here as it goes beyond a relational view, and enables effective interrogation as to why such categorisation perpetuates the corresponding stigma attached to PLHIV and poses a barrier to accessing legal benefits.

Within a broader legal and policy framework to address HIV, the state (through the NACO) constructs MSM, transgender persons, drug users, and sex workers as ‘high-risk’ groups in its National Guidelines for HIV/AIDS Testing.^101^ Such characterisation is not based on evidence and draws on societal perceptions of undesirability and uncleanness to justify targeting HIV intervention at these groups.^102^ For example, among Indian women, only 0.5 per cent of women with HIV are sex workers; on the other hand, almost 85 per cent of infections are from heterosexual sex within marriage or with primary partners.^103^

On one hand, characterisation of certain groups as ‘high-risk’ draws state attention to these marginalised groups and brings them into focus as subjects of treatment and welfare.^104^ Their visibility enables the government to consider the particular forms of discrimination that they face while formulating laws and policies, such that legal protections do not only cater to married, heterosexual couples.^105^

However, on the other hand, first, we argue that the use of ‘risk’ as a category of welfare is undesirable. It characterises the entire group as being defined in relation to HIV and could lead to discrimination on this basis. For example, sex workers are often denied medical treatment on the assumption that they *all* suffer from HIV.^106^ Here, HIV is an axis of discrimination not because of the presence of infection but due to the association of risk with certain groups. In fact, the HIV AIDS Bill, 2012, addressed this issue by including persons and groups who are *perceived to be vulnerable to HIV* as protected persons against discrimination.^107^ However, the current Act does not include such protection. This means that there could be unfavourable treatment in education, employment, housing, or health care against queer persons, trans people, and sex workers based on the *perception* that such persons have HIV. Given the lack of an overall anti-discrimination legislation in India, these categories would not have any legal protection against such discriminatory actions.

Secondly, another issue is that such categorisation of ‘risk’ results in stigma against certain kinds of activities (such as sex within the queer community and with sex workers or generally outside marriage). Such stigmatising notions of ‘risky and undesirable’ sexual activity are disguised as legitimate health concerns. On the flip side, married women are not considered to be high-risk as their sexuality and sexual activity are acceptable within society, and they are perceived as being in an acceptable monogamous sexual relationship. However, this schema is a red herring as not only does it reify sexual hierarchies of desirable and undesirable forms of sex, but also invisibilises married women and similar ‘respectable’ sexual categories in HIV prevention and control efforts.^108^

Thirdly and finally, not only is such stereotyping problematic in itself but stereotyping and stigma pose tangible barriers to accessing legal benefits and welfare measures. Empirical evidence underscores how stigma acts as a gateway barrier to every benefit the law formally secures. A pooled NFHS analysis (2005–2006 to 2015–2016) found that while overt social-distance stigma declined, the share of respondents afraid to disclose an HIV-positive status climbed from 31 to 37 per cent, a shift strongly associated with delayed testing and lower Antiretroviral Treatment (ART) uptake.^109^ Clinic-based and qualitative studies echo this pattern: fear of being recognised at an ART centre or having one’s status revealed deters women, queer persons, MSM, and sex workers from timely diagnosis.^110^ It also disrupts medication adherence, deepening the gap between legal promise and lived reality.^111^ Stereotyping and stigma thus cut both ways: the stigma attached to queer persons, MSM, and sex workers pre-emptively prevents testing as such groups do not want to be stereotyped and ridiculed; the flipside of ‘reputable groups’ such as married women not being categorised as ‘high-risk’ also inhibits health checkups, testing, and diagnosis of HIV. In practice, the right to free testing, treatment, maternity benefits, and insurance remain elusive for those who are stigmatised as risky, as well as those stereotyped as ‘reputable’ and ‘pure’.

The reproductive-justice lens identifies such stigma as a form of ‘reproductive oppression’^112^—a disciplinary force that governs who gets tested, accesses health care and welfare benefits, and is comfortable with sero-disclosure. Applying that insight to HIV governance yields two clear imperatives. One, risk-based categories must be decoupled from service eligibility; even NACO’s own Handbook on Prevention and Management of Stigma and Discrimination concedes that stigma ‘impedes the full enjoyment of legal rights’ and urges programme managers to treat its elimination as a core deliverable.^113^ Two, the policies classifying groups by the threshold of risk should re-evaluate the aim of such classification, and ensure that stigma and stereotyping do not prohibit effective access to welfare measures. These are essential steps if India’s HIV response is to satisfy reproductive justice’s demand for equal, stigma-free access to testing, health care, and welfare benefits.

### E. Concluding summary

In this section, the reproductive justice framework helps us see the limits of the rights-centric provisions of the Act by shifting the lens through which we construct a PLHIV as a subject in the law. The reproductive justice methodology enables us to see PLHIV as relational and sexual beings who are enmeshed in existing structures of marginalisation, and could also lie at the intersection of many of these structures. When viewed as such, the legal affirmation of individual rights is seen to fall short of creating sexual and reproductive empowerment for PLHIV and all their partners to be able to make more informed decisions for themselves. Finally, from a more holistic perspective, we see that there is a continuation of the stigmatic discourse of ‘risk’ that characterises PLHIV and various marginalised groups who are associated with HIV. It is necessary for us to know and understand these limits of the law and current legal discourse to be able to think beyond an individual rights-based framework to address these issues and to work towards sexual and reproductive justice for PLHIV. The reproductive justice framework uniquely helps interrogate and challenge larger structures—social, legal, institutional—that limit their agency. This is essential to advance justice not merely as recognition but as redistribution.

## V. CONCLUSION

This article has offered a critique of the HIV/AIDS Act from the perspective of reproductive justice. The use of this framework has enabled the authors to analyse whether the Act engages with the structural issues that cause stigma, discrimination, and lack of health care and treatment for PLHIV. In the first section, the article traced the features of the Act to show how it secures certain fundamental rights of PLHIV such as privacy, autonomy, and non-discrimination.

In the second section, the article located the Act in Indian jurisprudence on HIV/AIDS in judicial decisions relating to medical confidentiality and privacy, decriminalisation of Section 377, rights of queer persons, criminal provisions on transmission and sexual assault, and marriage and divorce. The article showed how the Act is quite progressive by using the language of privacy, informed consent, non-discrimination, and safe haven provisions. However, it also showed how there was non-engagement with the stigma attached to PLHIV in the context of sexual, marital, and familial relationships.

In the third section, the article used the reproductive justice framework to examine the gaps in the Act. The Act individualises the burden of precaution and primarily imposes the same only on the PLHIV, ignores the sexual freedom of their partners by not mandating disclosure in various cases, and limits the right to know to certain partners only. Generally, the Act fails to address partners’ right to make sexual and health care decisions for themselves. It also does not directly engage with the stigma attached to PLHIV as being sexual ‘risks’ to society, which is further exacerbated through AIDS control strategies employed by the state in India.

The reproductive justice framework requires two main things: first, taking a relational perspective towards addressing the sexual and reproductive-related concerns of PLHIV. Taking an individualist perspective towards decision making and controlling transmission both adopt a sex-negative approach and ignore the way PLHIV are enfolded into society, with families, partners, and caregivers. Second, there is a need to set up a strong medical and welfare infrastructure to support PLHIV medically, socially, and legally. The Act is inadequate on both these counts. Future reform efforts must consider embedding reproductive justice values within legislative drafting, judicial interpretation, and public health programming—ensuring that the law supports but does not eclipse broader cultural and institutional change.

In this vein, we recognise that some aspects such as sexual decision-making, attitudes of healthcare professionals, and stigma in society cannot be matters of legal change only. While anti-discrimination provisions and duties on healthcare professionals are necessary, there is a lot that leaks out of the bounds of the law—education, awareness, sensitisation, dialogue, and positive discourse. These can only arise out of activism, civil society and NGO engagement, progressive and inclusive sex education, free and wide-ranging testing, openness to dialogue about sex and sexual health, etc. Unless the Act’s legal framework is simultaneously bolstered by social and cultural change, the law will never be enough to bring about true reproductive justice.

To conclude, the Act has some significant provisions that address individual-level discrimination in the public sphere of employment, health care, education, housing, and isolation. However, it fails to address the intersectional structures that cause this discrimination, especially within sexual, marital, and familial relationships. A reproductive justice framework is required to identify the gaps in the law and supplement the privacy and liberal rights framework of the Act. That is the only way in which we can address HIV/AIDS in a more holistic and comprehensive manner.

## Footnotes

1 According to the Act, HIV/AIDS is a condition characterised by a combination of signs and symptoms, caused by Human Immunodeficiency Virus, which attacks and weakens the body’s immune system, making the HIV-positive person susceptible to life-threatening conditions. See HIV/AIDS Act, s 2(a) (‘2017 Act’).

2 Declaration of Commitment on HIV/AIDS, G.A. Res. S-26/2, U.N. Doc A/RES/S-26/2 (2001).

3 Loretta J Ross, ‘Conceptualizing Reproductive Justice Theory: A Manifesto for Activism’ in Loretta J Ross and others (eds), *Radical Reproductive Justice: Foundations, Theory, Practice, Critique* (Feminist Press 2017) 112–54.

4 Erika Derkas, ‘Retrofitting Choice: White Feminism and the Politics of Reproductive Justice’ in Loretta J Ross and others (eds), *Radical Reproductive Justice: Foundations, Theory, Practice, Critique* (Feminist Press 2017) 182–89; Loretta Ross, ‘Teaching Reproductive Justice: An Activist Approach’ in Olivia N Perlow and others (eds), *Black Women’s Liberatory Pedagogies* (Palgrave Macmillan 2018) 159–80; Johanna Gondouin, Suruchi Thapar-Bjorkert and Mohan Rao, ‘Dalit Feminist Voices on Reproductive Rights and Reproductive Justice’ (2020) 55 Economic & Political Weekly.

5 Introduction in Loretta J Ross and others (eds), *Radical Reproductive Justice: Foundations, Theory, Practice, Critique* (Feminist Press 2017) 7–20.

6 Robin West, ‘From Choice to Reproductive Justice: De-Constitutionalizing Abortion Rights’ (2009) 118 Yale Law Journal 1394.

7 Beverly Y Thompson, ‘Centering Reproductive Justice: Transitioning from Abortion Rights to Social Justice’ in Loretta J Ross and others (eds), *Radical Reproductive Justice: Foundations, Theory, Practice, Critique* (Feminist Press 2017) 168–81; see Gondouin, Thapar-Bjorkert and Rao (n 4) more generally for how abortions are contextualized within broader population control policies, and the decision to have children is impacted by several factors beyond the identity of the pregnant person/family.

8 HIV/AIDS Act 2017 (‘2017 Act’), ss 2(s) and 3.

9 2017 Act, s 2(d).

10 2017 Act, s 3(k).

11 2017 Act, s 4.

12 2017 Act, s 5.

13 2017 Act, s 6.

14 2017 Act, s 18(3).

15 In a case before the Punjab and Haryana High Court, the Court reaffirmed the duty of the State to provide medical treatment and requisite nutrition to PLHIV. See *Majinder Singh @ Johny v State of Punjab* CRM-M-54913/2019 (O&M). In other cases, the Court has granted bail to PLHIV on account of their HIV status, so they can receive adequate care, and has also ordered for their trial to be expedited under s 34(2) of the Act. See also *Rajan v State of Himachal Pradesh* 2022 SCC OnLine HP 6154 and *Aman Kumar v State of Himachal Pradesh* 2022 SCC OnLine HP 6529.

16 2017 Act, s 31.

17 2017 Act, s 10.

18 2017 Act, s 9.

19 2017 Act, s 34.

20 2017 Act, s 19.

21 2017 Act, ss 14 and 15.

22 2017 Act, ss 23 and 24.

23 This form of privacy has been defined and recognised by the Supreme Court of India in *KS Puttaswamy v Union of India* (2017) 10 SCC 1.

24 (1998) 8 SCC 296.

25 ibid [37].

26 ibid.

27
*Mr X v Hospital Z* (2003) 1 SCC 500.

28
*KS Puttaswamy* (n 23).

29 Compulsory Pre-Marital HIV Test and Other Measures Bill 2010.

30 Dipika Jain, *Against Mandatory Pre-Marital HIV Testing in India* (HRLN 2008).

31 ibid.

32 2017 Act, s 5.

33
*Lucy R D’Souza v State of Goa* AIR 1990 Bom 355.

34 ibid [8].

35 ibid.

36 Jayanth K Krishnan, ‘The Rights of the New Untouchables: A Constitutional Analysis of HIV Jurisprudence in India’ (2003) 25 Human Rights Quarterly 791.

37 ibid.

38 2017 Act, Preamble and s 3.

39 Indian Penal Code 1860, s 377.

40 R Ramasubban, ‘Political Intersections Between HIV/AIDS, Sexuality and Human Rights: A History of Resistance to the Anti-sodomy Law in India’ (2008) 3 Global Public Health 22.

41 ibid.

42 ibid.

43 Subir K Kole, ‘Globalizing Queer? AIDS, Homophobia and the Politics of Sexual Identity in India’ (2007) 3 Globalization and Health 8.

44 ibid.

45
*Naz Foundation v Government of NCT of Delhi* 2009 SCC OnLine Del 1762.

46 ibid.

47 ibid.

48 ibid [6].

49 ibid [72], [92], [93].

50 (2014) 1 SCC 1; Danish Sheikh, ‘The Quality of Mercy, Strained: Compassion, Empathy and Other Irrelevant Considerations in *Koushal v. Naz’* (2013) 6 NUJS Law Review 585.

51 2017 Act, s 22.

52 2017 Act, s 22, illustration (b).

53 (2018) 10 SCC 1.

54 ibid [505]–[514]; *National Legal Services Authority of India v Union of India* 2014 INSC 275 [48].

55 Saptarshi Mandal, ‘“Right to Privacy” in Naz Foundation: A Counter-Heteronormative Critique’ (2009) 2 NUJS Law Review 525.

56 ibid.

57 Immoral Traffic Prevention Act, 1956, ss 3–9.

58 This was the trend until the recent Supreme Court decision in *Budhadev Karmaskar v State of West Bengal* 2022 SCC OnLine SC 704, wherein the Supreme Court recognized and addressed both State violence and discrimination against sex workers.

59
*Sahyog Manila Mandal v State of Gujarat* 2004 SCC OnLine Guj 269.

60 ibid [20].

61 VAS Krishna and others, ‘Unheard Voices: Experiences of Families Living with HIV/AIDS in India’ (2005) 27 Contemporary Family Therapy 483.

62 Vivek Divan, ‘The HIV Act—better late than never’ (2018) 3 Indian Journal of Medical Ethics 35.

63 2017 Act, ss 9 and 10.

64
*Sabhajeet Maurya v State of NCT Delhi* 2020 SCC OnLine Del 1525.

65 ibid.

66 Lawrence Gostin and James Hodge, ‘Piercing the Veil of Secrecy in HIV/AIDS and Other Sexually Transmitted Diseases: Theories of Privacy and Disclosure in Partner Notification’ (1998) 5 Duke Journal Gender, Law and Policy 9.

67 Parsi Marriage and Divorce Act 1936, s 32(e); Dissolution of Muslim Marriages Act 1939, s 2(vi); Hindu Marriage Act 1955, s 13(1)(v); Special Marriage Act 1955, s 27(1)(f).

68
*A v B* 2013 SCC OnLine Mad 1345.

69
*P Ravikumar v Malarvizhi @ S Kokila* 2011 SCC OnLine Mad 244.

70 2017 Act, s 6.

71
*Mandeep Kaur v Dharminder Singh* 2014 SCC OnLine P&H 24154.

72 Barbara B Woodhouse and Katharine T Bartlett, ‘Sex, Lies, and Dissipation: The Discourse of Fault in a No-Fault Era’ (1994) 82 Georgetown Law Journal 2525; Ira M Ellman, ‘The Place of Fault in a Modern Divorce Law’ (1996) 28 Arizona State Law Journal 773.

73 2017 Act, s 3.

74
*Sabhajeet Yadav v State of UP* Writ A No 15103 of 2019 Allahabad High Court.

75 ibid.

76
*Union of India v Siva Lakshmi* WA No 595 of 2020 Madras High Court.

77 (1998) 8 SCC 296.

78 ibid [45].

79 Loretta J Ross and Rickie Solinger, *Reproductive Justice: An Introduction* (Regents of the University of California 2017) 1–8.

80 Asian Communities for Reproductive Justice, ‘A New Vision for Advancing Our Movement for Reproductive Health, Reproductive Rights and Reproductive Justice’ (2005) <https://forwardtogether.org/wp-content/uploads/2017/12/ACRJ-A-New-Vision.pdf> accessed 7 July 2024.

81 Swati Shah and others, ‘The Other Side of the Story: Intended Parents’ Surrogacy Journeys, Stigma and Relational Reproductive Justice’ (2022) 74 Health and Place 102769.

82 2017 Act, s 10.

83 2017 Act, s 10.

84 ibid.

85 Shipla N Patel and others, ‘Individual and Interpersonal Characteristics that Influence Male-Dominated Sexual Decision-Making and Inconsistent Condom Use Among Married HIV Serodiscordant Couples in Gujarat, India: Results from the Positive Jeevan Saathi Study’ (2014) 18 AIDS and Behaviour 1970.

86 Gauri Bhattacharya, ‘Sociocultural and Behavioural Contexts of Condom Use in Heterosexual Married Couples in India: Challenges to the HIV Prevention Program’ (2004) 31 Health and Education Behaviour 101.

87 ibid.

88 Rakhi Dandona and others, ‘High Risk of HIV in Non-Brothel Based Female Sex Workers in India’ (2005) 5 BMC Public Health 87.

89 Chaitanya Lakkimsetti, ‘“HIV Is Our Friend”: Prostitution, Biopower, and the State in Postcolonial India’ (2014) 40 Signs 201; Toorji Ghose and others, ‘Mobilizing Collective Identity to Reduce HIV Risk Among Sex Workers in Sonagachi, India: The Boundaries, Consciousness, Negotiation Framework’ (2008) 67 Social Science and Medicine 311.

90 Venkatesan Chakrapani and others, ‘HIV Status Disclosure and Condom Use Among HIV-Positive Men Who Have Sex with Men and *Hijras* (Male-to-Female Transgender People) in India: Implications for Prevention’ (2015) 14 Journal of HIV/AIDS and Social Services 26; Venkatesan Chakrapani and others, ‘Contextual Influences on Condom Use Among Men Who Have Sex With Men in India: Subjectivities, Practices and Risks’ (2013) 15 Culture, Health & Sexuality 938.

91 Deepika Ganju and Niranjan Saggurti, ‘Stigma, Violence and HIV Vulnerability Among Transgender Persons in Sex Work in Maharashtra, India’ (2017) 19 Cultural, Health and Sexuality 903.

92 See 2017 Act, s 29.

93 Krishna and others (n 61).

94 Srikant Sarangi, ‘Micropolitics of Disclosure, Stigma, and (Dis)trust Surrounding HIV/AIDS in India’ in Ivana Markova and Alex Gillespie (eds), *Trust and Distrust: Sociocultural Perspectives* (Information Age Publishing 2008).

95 Ross and Solinger (n 79) 1–8.

96 2017 Act, s 9.

97 2017 Act, s 2(p).

98 Ruth Dixon-Mueller and others, ‘Towards a Sexual Ethics of Rights and Responsibilities’ (2009) 17 Reproductive Health Matters 111.

99 Gostin and Hodge (n 66).

100 Asha Persson and others, ‘Bridging the HIV Divide: Stigma, Stories and Serodiscordant Sexuality in the Biomedical Age’ (2016) 20 Sexuality and Culture 197; Sameeksha Khare, ‘Love in the Time of HIV: When Stigma Couldn’t Stop These Couples’ (*The Quint*, 15 September 2018) <https://www.thequint.com/fit/love-in-the-time-of-hiv-when-stigma-couldnt-stop-these-couples#read-more> accessed 20 September 2022.

101 NACO, ‘National Guidelines for HIV Testing’ (2015) <http://www.naco.gov.in/sites/default/files/National_Guidelines_for_HIV_Testing_21Apr2016.pdf> accessed 21 September 2022.

102 Niranjan S Karnik, ‘Locating HIV/AIDS and India: Cautionary Notes on the Globalization of Categories’ (2001) 26 Science, Technology and Human Values 322.

103 ‘Gender Impact of HIV and AIDS in India’ (UNDP 2006) <https://core.ac.uk/download/pdf/43024213.pdf> accessed 20 September 2022.

104 Sunita Manian, *HIV/AIDS in India: Voices from the Margins* (Routledge 2017).

105 ibid.

106 Divan (n 62).

107 HIV/AIDS Bill 2012, s 2(v).

108 Bhattacharya (n 86).

109 Brian Chan, Venkatesan Chakrapani and Alexander C Tsai, ‘HIV-related Stigma Trends in the General Population of India During an Era of Antiretroviral Treatment Expansion, 2005-16’ (2020) 10 Journal of Global Health 020420.

110 Maria L Ekstrand, Shalini Bharat and Krishnamachari Srinivasan, ‘HIV Stigma is a Barrier to Achieving 90-90-90 in India’ (2018) 5 Lancet HIV e543; Shobha Shukla and Bobby Ramakant, ‘Stigma Has a Profound Impact on the Mental and Physical Health of Indian Women with HIV’ (2024, *AIDSMap)* <https://www.aidsmap.com/news/may-2024/stigma-has-profound-impact-mental-and-physical-health-indian-women-hiv> accessed 14 July 2025.

111 ibid.

112 Shah and others (n 81).

113 National AIDS Control Authority, ‘Handbook on Prevention and Management of Stigma and Discrimination Associated with HIV & AIDS’ (2022) <https://naco.gov.in/sites/default/files/NACO%20Stigma%20Handbook.pdf> accessed 14 July 2025.

